# Supplementary material for: Topological Lifshitz transitions and Fermi arc manipulation in Weyl semimetal NbAs
Source: Nat Commun. 2019 Aug 2;10:3478. doi: 10.1038/s41467-019-11491-4 (PMC6677823; doi:10.1038/s41467-019-11491-4)
Supplement: Supplementary file 1 — Supplementary Information [file 41467_2019_11491_MOESM1_ESM.pdf]

Topological Lifshitz Transitions and Fermi Arc Manipulation in Weyl Semimetal NbAs

Yang et al.

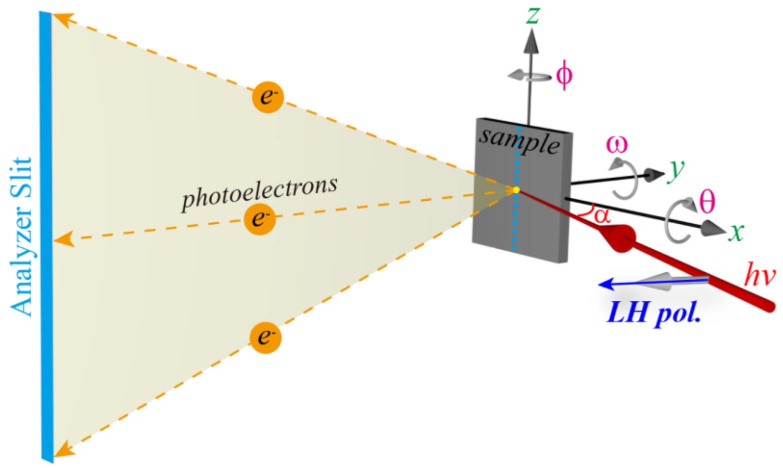

Supplementary Figure 1 | Illustration of the ARPES experiment configuration. When the polar angle ϕ is zero, the angle between the photon beam and the sample surface (α) is 40 degrees.

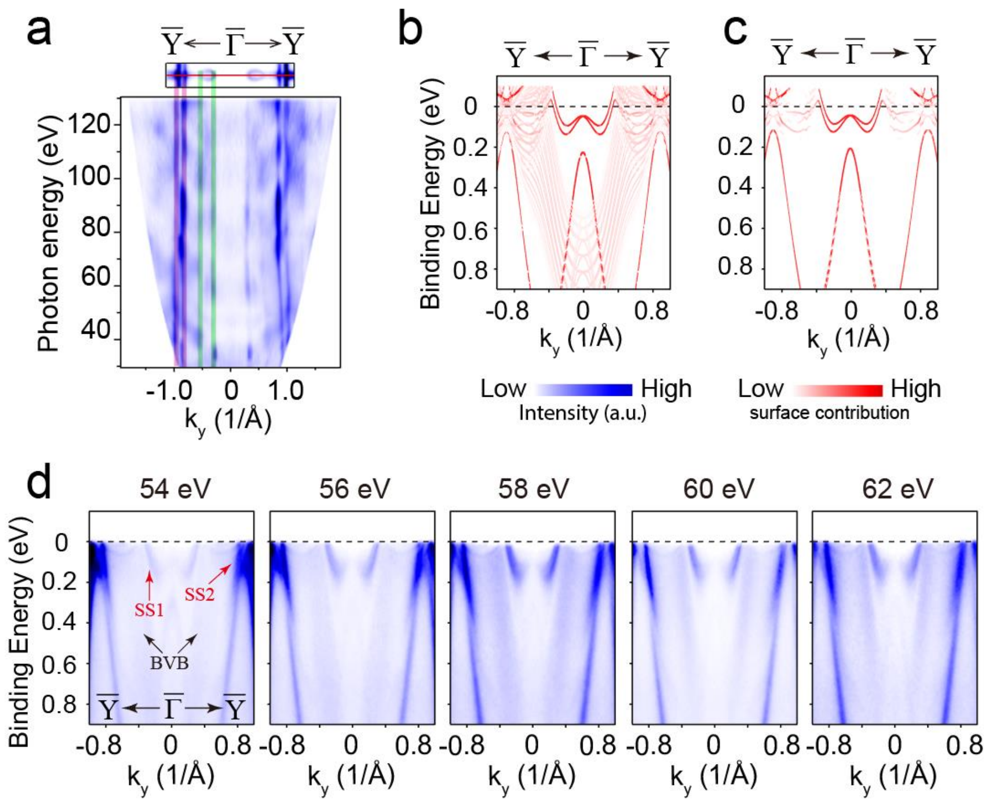

Supplementary Figure 2 | Photon-energy dependent measurements on pristine NbAs. a, The photoemission intensity map at E_F along the $\bar{Y}-\bar{\Gamma}-\bar{Y}$ (see the red line marked on the FS portion in the upper panel) for broad photon energy range from 30 eV to 130 eV, showing non-dispersive vertical lines for the surface states. **b-c**, calculated band dispersions along the $\bar{Y}$-$\bar{\Gamma}$-$\bar{Y}$ direction, showing the surface states which agree with ARPES measurements in **d**. The red color scale is proportional to the surface state component. Note that **b** includes both the bulk and surface states, while **c** mainly consists of surface states for clarity. **d**, Detailed band dispersions along the $\bar{Y}$-$\bar{\Gamma}$-$\bar{Y}$ direction from different photon energies show unchanged (surface state) dispersions.

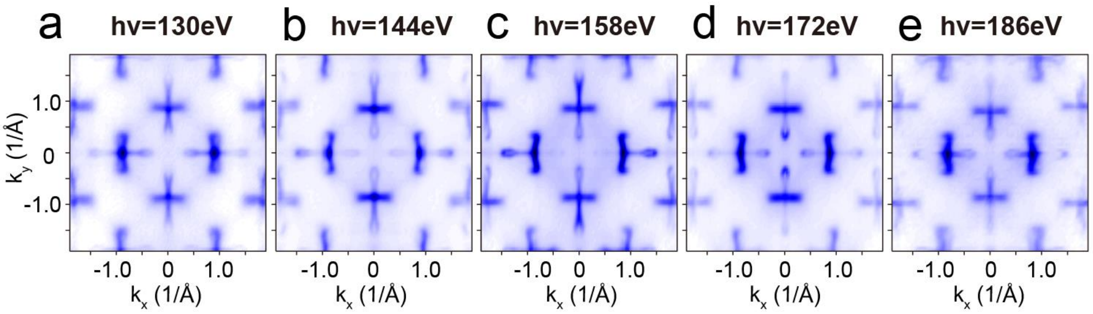

Supplementary Figure 3 | Fermi surfaces measured with a broad energy range of photons. a-e, Fermi surface maps measured with 130, 144, 158, 172 and 186 eV photons, respectively. These maps are mirror symmetrized to reflect the symmetry of the crystal.

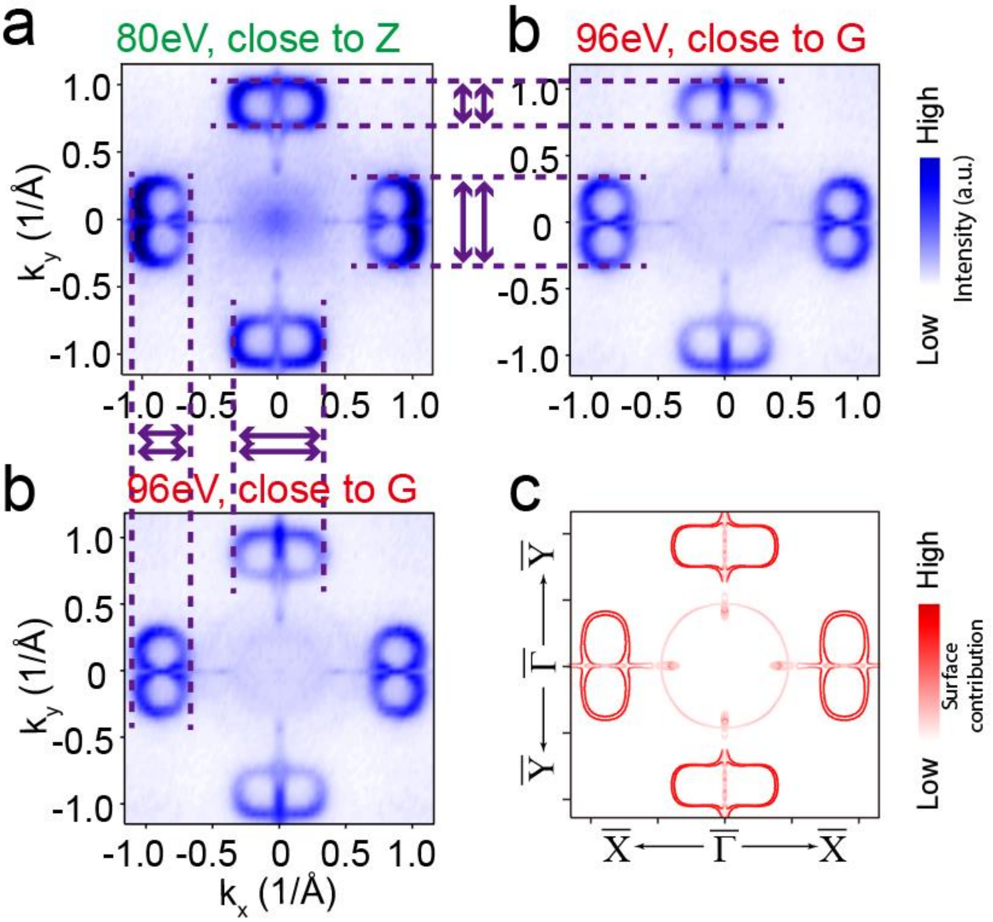

Supplementary Figure 4 | Surface origin of figure-8-like Fermi surface. a, Measured Fermi surface with 80-eV photons which is close to the bulk *Z* point. **b**, Measured Fermi surface with 96-eV photons which is close to the bulk Γ point. Purple dotted lines are used to compare the size of these figure-8-like features measured at 80 and 96 eV. **c**, calculated surface Fermi surface of the K-decorated NbAs.

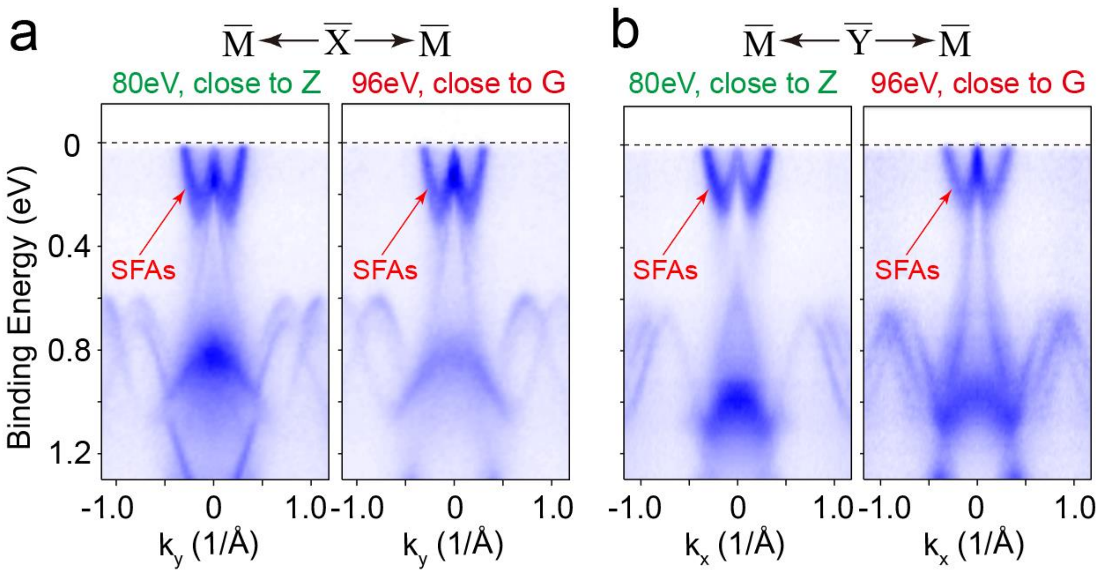

Supplementary Figure 5 | Measured dispersions with 80eV and 96eV photons. Note that 80-eV and 96-eV are close to the bulk Z and Γ points, respectively.

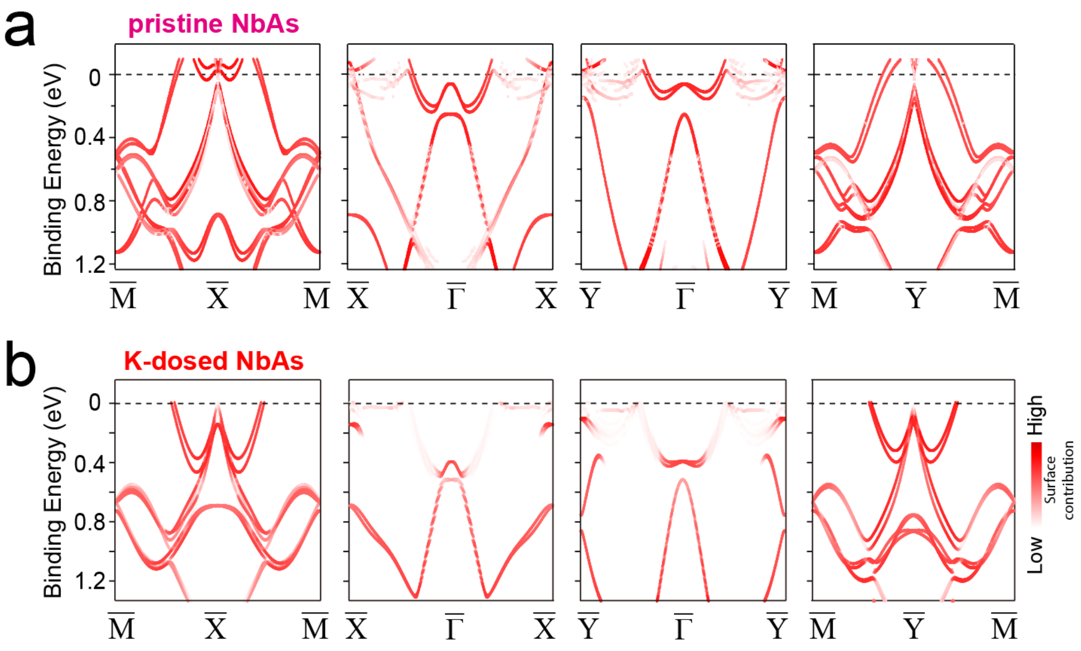

Supplementary Figure 6 | Comparison of calculated high-symmetry band dispersions. Pronounced differences between them can be clearly found.

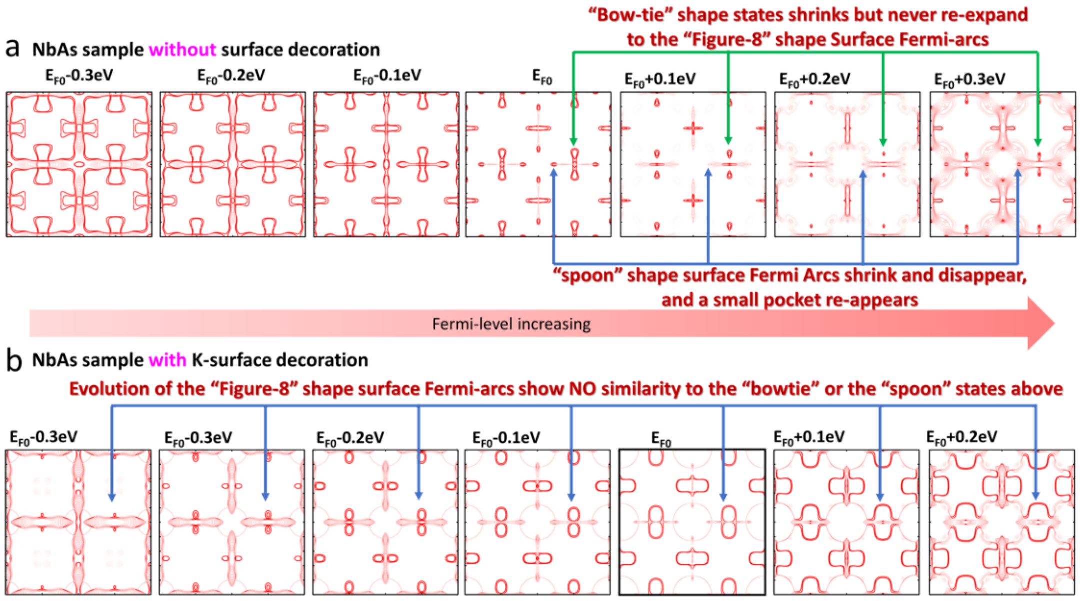

Supplementary Figure 7 | Direct comparison of Fermi contours. Apparently, one cannot find one constant-energy contour of the K-dosed that matches any one of the pristine.

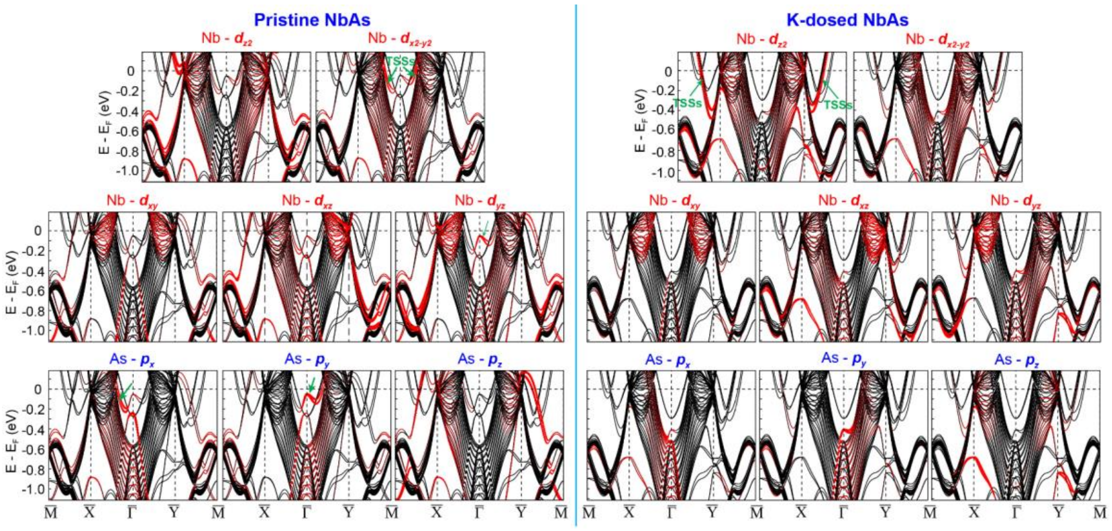

Supplementary Figure 8 | Orbital component calculations. Calculated high-symmetry band dispersions projected on Nb d_{z2}, $d_{x2\text{-}y2}$, d_{xy}, d_{yz}, d_{xz}, and As p_x, p_y, p_z orbitals of the pristine and K-dosed NbAs. Black curves are bulk states, dispersions marked by red circles are projected surface states with different orbital contributions (bigger red circles represent stronger contribution). While the TSS of the pristine surface harbours a complicated multi-orbital contributions from both As and Nb, the TSS of K-decorated is dominated by Nb d_{z^2} orbital.

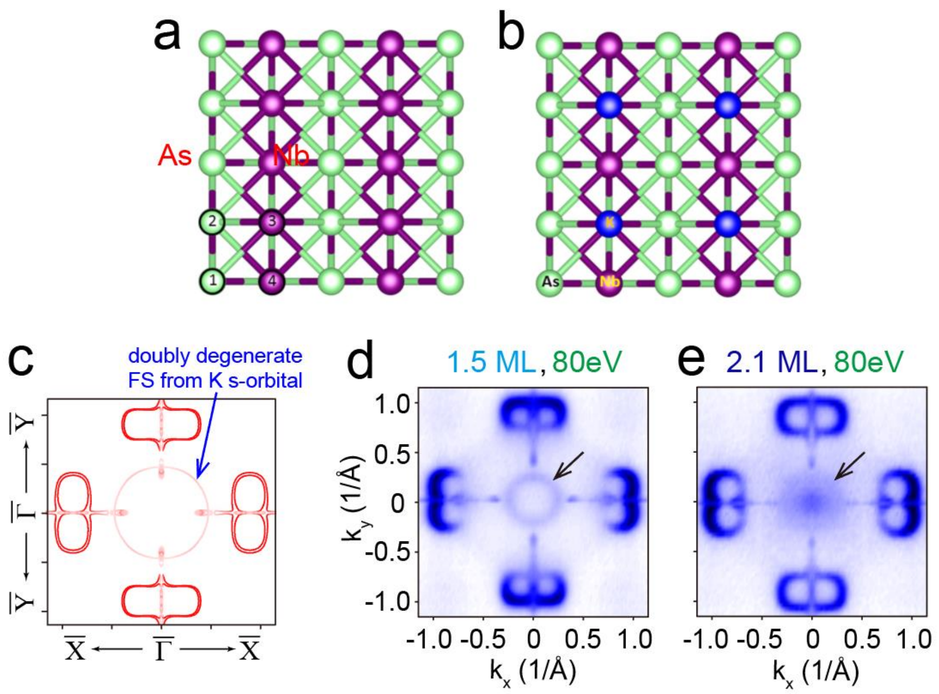

Supplementary Figure 9 | K *s*-band. a, Four possible locations for K atoms on the As-terminal are labeled as 1, 2, 3, 4, respectively. **b**, The stable state with the minimum energy. **c**, Projected FS of K-decorated NbAs from top layers of NbAs. Circle-like FS (enclosing the $\bar{\Gamma}$ point) marked by the black arrow originates from the K *s*-orbital. **d-e**, Measured FSs of 4th K-decorated (1.5 ML) and 5th K-decorated (~2.1 ML, see discussion in Supplementary Note 5 below) with 80 eV photons.

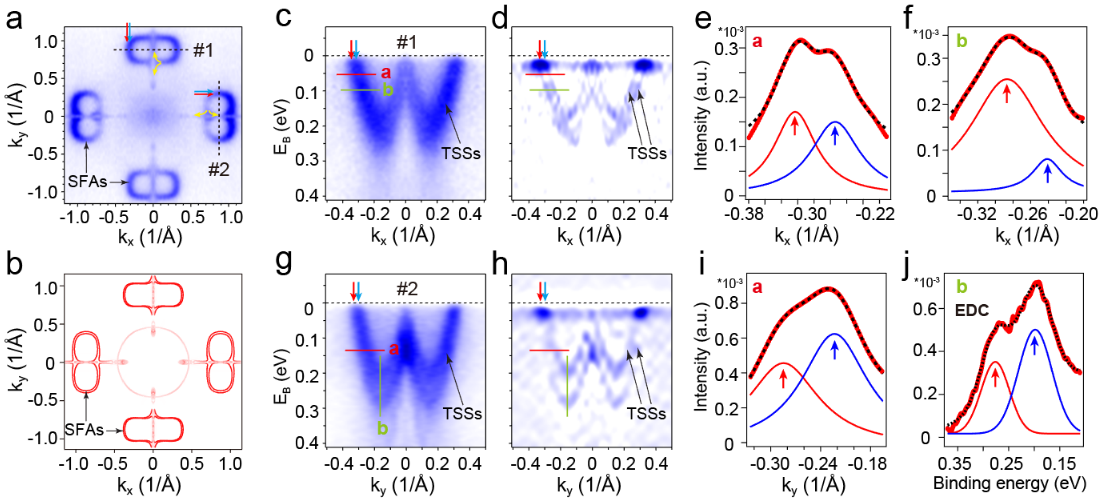

Supplementary Figure 10 | Examining the fine structure of Figure-8-like feature. a-b, Direct comparison of measured and calculated Fermi surface, showing nice agreement. **c-f** are band dispersions along Cut #1, second-derivative plot, two MDCs and their fitting results with two Lorentz peaks plus a linear background, respectively. The dotted black curves are the fitted. **g-j** are band dispersions along Cut #2, second-derivative plot, one MDC/EDC and its fitting results with two Lorentz/Gauss peaks plus a linear background, respectively. The dotted black curves are the fitted.

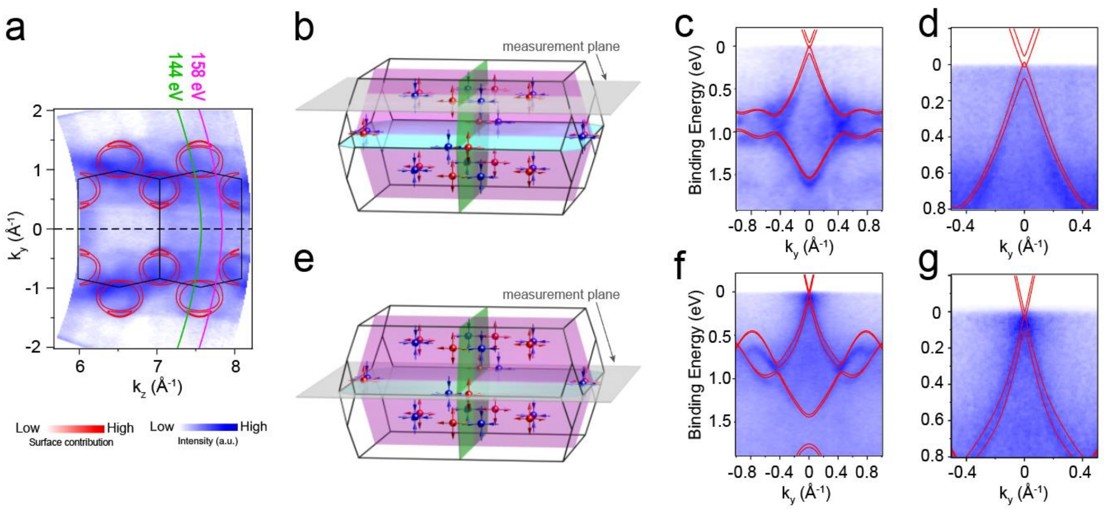

Supplementary Figure 11 | Bulk band structure of NbAs. a, photoemission intensity plot (integration window is [E_F-100meV, E_F]) with overlaid calculations of bulk states (red curves). Note that although surface states are highly suppressed to enhance the bulk states in the photoemission intensity, some surface states remain to be weak features (not captured by calculations of bulk states), as the probe depth in our measurements is around 1.0 nm. With 158 eV and 144 eV photons, we can reach W2 and W1 Weyl points, respectively. **b**, Schematic illustration of the measurement plane in 3D bulk BZ when using 158 eV photons. **c-d**, bulk band dispersion across W2 Weyl points: broadband dispersion and a zoomed-in plot across the Weyl points along k_y direction. Calculations of bulk states (red curves) well reproduce measured bulk states. **e-g**, same as **b-d**, but across W1 Weyl points using 144eV photons.

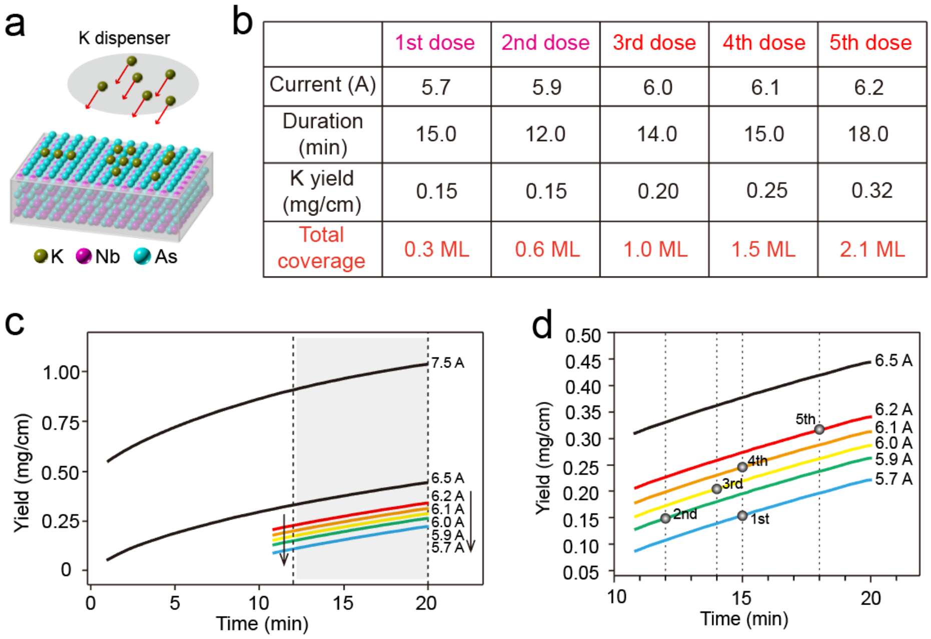

	1st dose	2nd dose	3rd dose	4th dose	5th dose
Current (A)	5.7	5.9	6.0	6.1	6.2
Duration (min)	15.0	12.0	14.0	15.0	18.0
K yield (mg/cm)	0.15	0.15	0.20	0.25	0.32
Total coverage	0.3 ML	0.6 ML	1.0 ML	1.5 ML	2.1 ML

Supplementary Figure 12 | Details of K dosing. a, Schematic of K dosing by a commercial Saes K dispenser. **b**, Detailed dosing parameters and calculated amount of accumulated K atoms on the sample surface. **c**, Performance curves of K dispenser. Black curves (7.5A and 6.5A) are directly reproduced from the technical book of the commercial SAES K-dispenser (Alkali Metals Dispensers – SAES Getters)[5], and curves with colors are extrapolated performances curves with different currents. In (ii), the K yield of each dose is marked.

Supplementary Note 1:

Determine the surface states of pristine and surface decorated NbAs

ARPES is an experimental method that can directly visualize the electronic structures of solids based on the photoelectric effect, where an electron inside the sample under investigation can absorb an incident photon (whose energy is larger than the work function) and then escape the sample as a photoelectron. The kinetic energy and momentum of the photoelectron are then measured to give the information of the initial electronic band structures of the sample[1,2].

In ARPES measurements, the in-plane electron momentum ($k_{||}$, parallel to sample surface) can be naturally determined by the momentum conservation of photoelectrons; while determining the out-of-plane momentum component (k_z) and band dispersion along k_z requires a series of ARPES measurements performed at different photon energies[1,2].

Based on the free-electron final state approximation with a potential parameter V_0 (the inner potential) describing the energy difference of photoelectrons before and after leaving the crystal surface, we can derive the k_z as:

$$k_z = \frac{\sqrt{2m_e^*(E_k \cos^2\theta + V_0)}}{\hbar}$$

where θ is the emission angle and E_k is the kinetic energy of the emitted electron, which satisfies:

$$E_k = h\upsilon - w - E_B$$

where $h\upsilon$ is the photon energy, w is the work function of the sample and E_B is the electron binding energy.

Therefore, photon-energy dependent ARPES measurements can probe the electronic structure with different k_z values, thus can be used to discriminate the surface electronic states (which do not disperse along k_z direction) from the bulk ones (which usually show dispersion along k_z direction)[1,2].

In this work, the ARPES measurements were performed at beamline I05 of the Diamond Light Source (DLS) by Scienta R4000 analyzers. The angle resolution was $\leq 0.2°$ and the overall energy resolutions was ≤ 15 meV. The measurement geometry and the light polarization of our ARPES experiments are shown in Supplementary Figure 1, where the sample sits on a 6-axis manipulator with three translational and three rotational degrees of freedom. The analyzer slit is vertical (along the z-axis) and the photon polarization used was linearly horizontal (LH, i.e. the electric field lies in the x-y plane, see Supplementary Figure 1). The Fermi surface mapping was obtained by rotating the sample angle ϕ (polar angle, see Supplementary Figure 1); and the broad photon energy range (30 – 180 eV) was used during the photon-energy dependent measurement to access the different k_z momenta.

In the main text, we have presented the general band structure of pristine NbAs. The comparison with our *ab initio* calculations (Fig. 3) gives a nice agreement which reveals that the bowtie- and spoon-like features are of surface origin and confirms the existence of surface Fermi arcs. To further verify this experimentally, we carried out systematic photon-energy dependent measurements to study the k_z variation of the band structure.

Supplementary Figure 2a shows the photoemission intensity map at E_F along the k_y direction with different photon energy. The vertical lines from the bowtie FS across different photon energy (therefore different k_z) clearly show their surface origin. These surface bands can also be clearly seen in the calculation in Supplementary Figure 2b and 2c. In addition, Supplementary Figure 2d further illustrates band dispersion details along $\bar{Y}$- $\bar{\Gamma}$- $\bar{Y}$ direction measured with photons from 54 to 62 eV; the nice agreement with the calculation in Supplementary Figure 2b and 2c, and no resolvable changes of SS1 and SS2 observed by varying the photon energy further confirm their surface origin.

In addition to the features around $\bar{Y}$ point, FSs at different photon energies are also acquired to further examine the surface origin of bowtie and the spoon FSs both around $\bar{X}$ and $\bar{Y}$ points (Supplementary Figure 3). Clearly, the FS maps of these pockets remain the same under different photon energies (from 130 eV to 186 eV), again confirming the surface natures of the bowtie- and spoon-like FS pockets.

In the main text, we have presented the general band structure of K-decorated NbAs. The comparison with our *ab initio* calculations (Fig. 3) gives a nice agreement which reveals that figure-8-like feature actually purely consists of long SFAs. To further verify the surface nature of this feature experimentally, we carried out photon-energy dependent measurements.

Supplementary Figure 3 shows the Fermi surfaces measured with 80 and 96 eV photons, respectively. Note that 80 eV is close to the bulk Z point, and 96 eV is close to the bulk Γ point, which is similar to other TMM family members like TaAs reported[3]. If the Fermi surfaces mainly consist of bulk states, Fermi surfaces acquired at 80 and 96 eV would be evidently different. However, both maps apparently exhibit figure-8-like features, and their sizes are in excellent agreement (overlaid dotted lines are used to compare their sizes) which can also be seen from high-symmetry dispersions in Supplementary Figure 5. Besides, these features are well reproduced by our *ab initio* calculations of surface states (see Fig. 2b of the main text and Supplementary Figure 7 below), which further confirms the figure-8-like feature is of surface origin.

Supplementary Note 2:

Comparison of calculated band structures of pristine and K-decorated NbAs surfaces

In the main text, we have shown the calculation results of pristine and K-decorated NbAs surface, both of which exhibit excellent agreements with ARPES data. Here, we present more detailed calculation results and show why this difference cannot be explained by rigid shift of E_F.

Supplementary Figure 6 and 7 display calculated high-symmetry dispersions and constant-energy contours of pristine and K-decorated NbAs surface side-by-side for comparison, respectively. Note that the color scale represent the surface projection of bands, i.e. the bold red curves represent the surface states while the faded red curves represent bands with strong bulk origin.

From the first principle calculations, it is clear that the Fermi-surface evolution caused by the lifting of the Fermi-level (i.e. the rigid band shift) cannot explain the dramatic change of the Fermi-surfaces we observed, which shows that the effect of the surface K-decoration is not simple (n-type) charge doping, but rather a modification of the surface environment with associated new bands (i.e. surface Fermi-arcs) formations.

For NbAs without surface decoration, if we shift up the Fermi-level, both the spoon-like surface Fermi-arcs and bow-tie-like trivial states will shrink (see Supplementary Figure 7 above), but none will evolve into the new figure-8 shape surface Fermi-arcs we observed after the K-surface decoration. On the other hand, the figure-8 shape surface Fermi-arcs naturally occurs from the K-decorated sample, as the first principle calculations shown in Supplementary Figure 7b. The new Fermi-surfaces geometry and topology in Supplementary Figure 7b is completely different from those in Supplementary Figure 7a. One cannot find a constant-energy contour of the pristine surface that corresponds to any constant-energy contour (even at different binding energies) from the K-decorated surface, meaning that the rigid band shift cannot explain the band structure differences of the pristine and K-decorated surfaces. Thus, the dramatic change of surface Fermi-arcs before (spoon-like shapes) and after (figure-8-like shapes) shows a topological Lifshitz transition – which is different from the regular Lifshitz transition showing the Fermi-surface topology change that is caused by the change of Fermi level.

Further, we performed *ab-initio* calculations of orbital contributions to near-E_F band structures of the pristine and K-decorated NbAs, as shown in Supplementary Figure 8. With these calculations, complicated band structures are decomposed into different individual orbital contributions, which could highly facilitate the investigations on the band structure evolution

across the K-dose induced transition. For the pristine, the TSSs is mainly contributed by As p_x, p_y, and Nb $d_{x^2\text{-}y^2}$ (and some few contributions from other orbitals like d_{yz}). By contrast, the TSSs of K-decorated is simply dominated by Nb d_{z^2} orbital. Moreover, bands stemming from each of these orbitals, e.g. Nb d_{z^2}, changes dramatically which cannot be explained simply with rigid-band shift.

In our *ab initio* calculation, K-decorated NbAs surface is treated as the cleaved NbAs surface covered with a monolayer K atoms (see Supplementary Figure 9a, b). We have tried four different high-symmetry locations of K on the As-terminated side. By checking the total energy of all four possible lattice configuration, the ground state is shown in Supplementary Figure 9b, where the distance between K and substrate NbAs is around 3.3Å. Interestingly, such a surface K-layer not only results in the figure-8 shape SFAs as discussed in the main text, but also gives an additional circle-like surface FS centered at the $\bar{\Gamma}$ point (marked by the blue arrow in Supplementary Figure 9c) originating from the K *s*-orbital, which is doubly degenerated (thus does not affect the non-trivial topology of NbAs SFAs) and away from the SFAs around the $\bar{\text{X}}$ and $\bar{\text{Y}}$ points.

This unique feature can be used to check the validity of the model we use in the *ab initio* calculations. By comparing to the ARPES experiments, we indeed observed a circle-like FS around the $\bar{\Gamma}$ point (see Supplementary Figure 9d and 9e, indicated by the black arrow) in our measurements. If we keep increasing K-doping, this circle-like FS becomes bigger but blurry, as excess K dosing unavoidably introduces disorders on the sample surface and destroys this topologically trivially surface FS. Remarkably, however, even when the circle-like FS disappears due to the excess dosage of K atoms, the figure-8-like SFAs remains, clearly showing its robustness due to its topological origin.

Supplementary Note 3:
Fine structures of the Figure-8-like SFAs

In the Fig. 4 of the main text, we have illustrated fine structures of Figure-8 FS with improved data quality (whose K dosage is 1.8 ML, slightly smaller than that of the K-dosed data in Figs. 2 and 3). Herein, for K-dosed data presented in Figs. 2 and 3, fine structures of Figure-8-like FS can also be resolved, by means of second derivative and MDC/EDC analysis. From raw data, one can clearly see the broad feature splits into two branches near $k_x/k_y = 0$ regions, as guided by yellow arrows in Supplementary Figure 10. Second-derivative analysis on band dispersions along #1 ($\bar{\text{M}}$ - $\bar{\text{Y}}$ - $\bar{\text{M}}$) and #2 ($\bar{\text{M}}$ - $\bar{\text{X}}$ - $\bar{\text{M}}$), reveals four band-crossings of the TSSs contributing to two SFAs of the figure-8 (Supplementary Figure 10d, h). Moreover, several representative MDCs/EDC from dispersions cutting through the figure-8, show two-peak

structures, as plotted in Supplementary Figure 10e, f, i, j. Further, these MDCs/EDC can be nicely fitted with two Lorentz/Gauss peaks and a linear background (note that dotted black line is the fitted curve). These results, again, reveal that Figure-8-like FS is purely formed by two long SFAs.

Supplementary Note 4:
Bulk Weyl dispersions

Besides the topological Lifshitz transition of SFAs, bulk Weyl nodes and their connectivity with SFAs (with/without K-decoration) in NbAs are also investigated. In Supplementary Figure 11a, the constant-energy contour in the $k_y - k_z$ plane ($k_x = 0$) (integration energy window: [E_F-100 meV, E_F]), obtained by the by photon energy dependent ARPES measurements shows strong k_z dispersions, in agreement with our *ab-initio* calculations of bulk states (red curves), confirming their bulk state origin.

With band structures along k_z being measured, we can further measure bulk Weyl dispersions across W1 and W2 Weyl nodes (theoretically predicted to be located at k_z planes of $k_z = 0$ and $k_z = \pm 1.17\pi/c$, respectively), which can be assessed by photons of 144 eV and 158 eV respectively as marked by the green and magenta curves in Supplementary Figure 11a. Supplementary Figure 11c, d, and 11f, g display bulk Weyl dispersions across W2 and W1 with overlaid calculation results (red curves), respectively. These bands near E_F clearly disperse linearly and are nicely reproduced by our calculations, thus confirming the existence of bulk Weyl nodes of NbAs. These results are also in good agreement with previous work on NbAs[4].

As shown in the Fig. 2 of the main text, SFAs essentially connects projections of bulk Weyl points, confirming the topological Weyl semimetal nature of NbAs. Moreover, after the topological Lifshitz transition of SFAs induced by K-decoration, SFAs remain tied to projections of bulk Weyl points (Fig. 2 in the main text), which confirms the topological robustness of topological Weyl semimetals.

Supplementary Note 5:
Estimate of K dosage

Potassium (K) atoms were *in situ* evaporated onto cleaved (001) surface of NbAs by the commercial SAES potassium dispenser (Supplementary Figure 12a), under an ultrahigh vacuum of 2.0×10^{-10} Torr at measurement temperature (10 K). The dosage of K atoms is determined by the dispenser current and the evaporation time (Supplementary Figure 12c, d). Therefore, by controlling the dispenser current and the evaporation time, we were able to

control the dosage of K atoms, and the detailed evaporation parameters used in our experiments are presented in Supplementary Figure 12b.

The amount of K atoms docked on the sample surface at each dose can be estimated as the following: the K flux (*F*, per unit area) near the sample is approximately given by $F = \frac{X*Y}{M} * N_A \div (2\pi R^2)$, where *X*, *Y*, *M*, N_A, and *R* are the active length of the dispenser (the length of the K filament), K (mass) yield of unit length from the dispenser (see Supplementary Figure 12), molar mass of K atoms, Avogadro's constant, and the distance between the dispenser and the sample, respectively. Finally, we can get the dosage (*D*) in the unit of ML as: $D = \frac{F}{d} * k$, where *d* is the number density for 1- ML of K coverage according to the calculation model (see Supplementary Figure 9), and *k* is the sticking coefficient of K atoms.

Supplementary References:

1. Damascelli, A., Hussain, Z. & Shen, Z. X. Angle-resolved photoemission studies of the cuprate superconductors. *Rev. Mod. Phys.* **75**, 473-541 (2003).
2. Chen, Y. L. Studies on the electronic structures of three-dimensional topological insulators by angle resolved photoemission spectroscopy. *Front. Phys*. **7**, 175-192 (2012).
3. Yang, L. X. *et al*. Weyl semimetal phase in the non-centrosymmetric compound TaAs. *Nat. Phys.* **11**, 728-732 (2015).
4. Xu, S. Y. *et al*. Discovery of a Weyl fermion semimetal and topological Fermi arcs. *Science.* **349**, 613-617 (2015).
5. Succi, M., Canino, R. & Ferrario, B. Atomic absorption evaporation flow rate measurements of alkali metal dispensers. Vacuum **35**, 579-582 (1985).
